# P-1913. Characteristics, Clinical Management, and Outcomes of Leukemia, Lymphoma and Multiple Myeloma Patients Hospitalized with a Primary Diagnosis of COVID-19: Insights from Hospitals across the United States

**DOI:** 10.1093/ofid/ofae631.2074

**Published:** 2025-01-29

**Authors:** Essy Mozaffari, Aastha Chandak, Chidinma Chima-Melton, Andre Kalil, Mark Berry, Heng Jiang, Michele Bartoletti, Paul Loubet

**Affiliations:** Gilead Sciences, Foster, California; Certara, New York, New York; University of California Los Angeles, Los Angeles, California; University of Nebraska Medical Center; Gilead Sciences, Inc., Foster City, California; Certara, New York, New York; Department of Biomedical Sciences, Humanitas University, Pieve Emanuele (Italy); IRCCS Humanitas Research Hospital, Rozzano (Italy), Rozzano, Lombardia, Italy; CHU de Nîmes, Nimes, Languedoc-Roussillon, France

## Abstract

**Background:**

Immunocompromised (IC) patients remain at high risk of hospitalizations, complications, and mortality due to COVID-19. However, limited information is available on specific IC conditions. We describe patient characteristics, clinical management of patients with leukemia, lymphoma or multiple myeloma hospitalized for COVID-19.

**Table 1:**
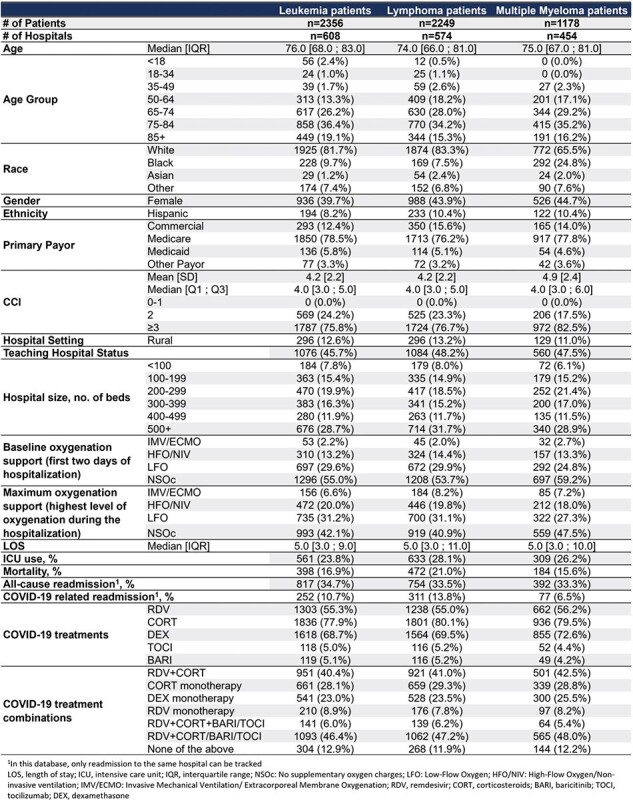
Characteristics of Leukemia, Lymphoma and Multiple Myeloma patients hospitalized for COVID-19

**Methods:**

Using the PINC AI Healthcare database, patients with leukemia, lymphoma or multiple myeloma hospitalised during Jan’22-Dec’23 with a primary discharge diagnosis of COVID-19 were examined. Patient characteristics were described for the three IC conditions. Utilization of COVID-19 treatments by supplemental oxygen requirements and outcomes of in-hospital mortality, all-cause and COVID-19 related readmission were assessed.

**Figure 1:**
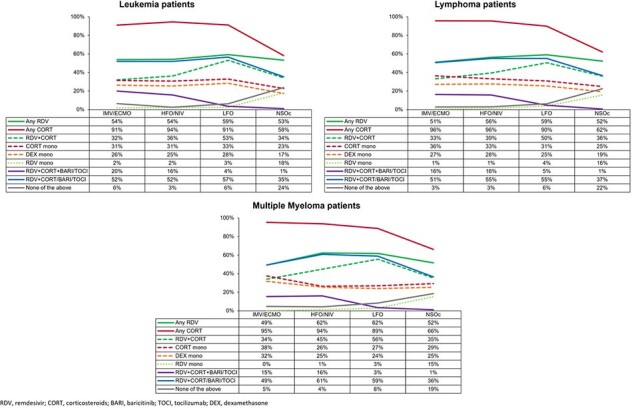
COVID-19 treatments by supplemental oxygen requirements during the hospitalization

**Results:**

During the study period, 2356 leukemia, 2249 lymphoma and 1178 multiple myeloma patients were hospitalized with a primary diagnosis of COVID-19. During the hospitalization, around a third required low-flow oxygen, one-fifth high-flow oxygen/non-invasive ventilation and 7-8% invasive mechanical ventilation. Further, mortality rates were 17%, 21% and 16% and COVID-19 related readmission rates were 11%, 14% and 7% for the leukemia, lymphoma and multiple myeloma patients, respectively (Table 1).

Half of the patients received remdesivir across all levels of oxygenation and >90% received corticosteroids among those requiring supplemental oxygen. Further, use of corticosteroid monotherapy was observed in a third of the patients requiring any supplemental oxygen, and in a quarter not requiring supplemental oxygen. A quarter of the patients without supplemental oxygen did not receive any COVID-19 treatment (Fig 1).

**Conclusion:**

The study highlights the high mortality and readmission rates faced by the high-risk IC patients with underlying conditions of leukemia, lymphoma or multiple myeloma hospitalized for COVID-19. Almost half did not receive antiviral treatment with remdesivir despite being hospitalized with a primary diagnosis of COVID-19, while a third received corticosteroid monotherapy contradicting guideline recommendations. Further, about a quarter without supplemental oxygen did not receive any COVID-19 treatment despite established effectiveness of remdesivir in this group.

**Disclosures:**

Essy Mozaffari, PharmD, MPH, MBA, Gilead Sciences, Inc.: Employee|Gilead Sciences, Inc.: Stocks/Bonds (Public Company) Aastha Chandak, PhD, Gilead Sciences Inc.: My organization (Certara) was contracted by Gilead to conduct this study Chidinma Chima-Melton, MD, Gilead Sciences: Advisor/Consultant Mark Berry, PhD, Gilead Sciences, Inc.: Employee|Gilead Sciences, Inc.: Stocks/Bonds (Public Company) Heng Jiang, MS, MPH, Gilead Sciences Inc.: My organization (Certara) was contracted by Gilead to conduct this study Michele Bartoletti, MD, PhD, Advan pharma: Advisor/Consultant|Advan pharma: Honoraria|Biomereux: Honoraria|Gilead: Advisor/Consultant|Gilead: Honoraria|Infectopharma: Advisor/Consultant|Msd: Advisor/Consultant|Msd: Grant/Research Support|Msd: Honoraria|Pfizer: Honoraria Paul LOUBET, MD, PhD, Astrazeneca: Advisor/Consultant|Gilead: Advisor/Consultant|Moderna: Advisor/Consultant|Pfizer: Advisor/Consultant

